# Frequent amplification of receptor tyrosine kinase genes in well-differentiated/ dedifferentiated liposarcoma

**DOI:** 10.18632/oncotarget.14652

**Published:** 2017-01-14

**Authors:** Naofumi Asano, Akihiko Yoshida, Sachiyo Mitani, Eisuke Kobayashi, Bunsyo Shiotani, Motokiyo Komiyama, Hiroyuki Fujimoto, Hirokazu Chuman, Hideo Morioka, Morio Matsumoto, Masaya Nakamura, Takashi Kubo, Mamoru Kato, Takashi Kohno, Akira Kawai, Tadashi Kondo, Hitoshi Ichikawa

**Affiliations:** ^1^ Division of Rare Cancer Research, National Cancer Center Research Institute, Chuo-ku, Tokyo 104-0045, Japan; ^2^ Department of Orthopaedic Surgery, Keio University School of Medicine, Shinjuku-ku, Tokyo 160-8582, Japan; ^3^ Department of Pathology and Clinical Laboratory, National Cancer Center Hospital, Chuo-ku, Tokyo 104-0045, Japan; ^4^ Department of Clinical Genomics, National Cancer Center Research Institute, Chuo-ku, Tokyo 104-0045, Japan; ^5^ Department of Musculoskeletal Oncology, National Cancer Center Hospital, Chuo-ku, Tokyo 104-0045, Japan; ^6^ Division of Genetics, National Cancer Center Research Institute, Chuo-ku, Tokyo 104-0045, Japan; ^7^ Department of Urology, National Cancer Center Hospital, Chuo-ku, Tokyo 104-0045, Japan; ^8^ Division of Translational Genomics, National Cancer Center-Exploratory Oncology Research and Clinical Trial Center, Chuo-ku, Tokyo 104-0045, Japan; ^9^ Department of Bioinformatics, National Cancer Center Research Institute, Chuo-ku, Tokyo 104-0045, Japan; ^10^ Division of Genome Biology, National Cancer Center Research Institute, Chuo-ku, Tokyo 104-0045, Japan

**Keywords:** liposarcoma, well-differentiated liposarcoma, dedifferentiated liposarcoma, next-generation sequencing, receptor tyrosine kinase

## Abstract

Well-differentiated liposarcoma (WDLPS) and dedifferentiated liposarcoma (DDLPS) are closely related tumors commonly characterized by *MDM2/CDK4* gene amplification, and lack clinically effective treatment options when inoperable. To identify novel therapeutic targets, we performed targeted genomic sequencing analysis of 19 WDLPS and 37 DDLPS tumor samples using a panel of 104 cancer-related genes (NCC oncopanel v3) developed specifically for genomic testing to select suitable molecular targeted therapies. The results of this analysis indicated that these sarcomas had very few gene mutations and a high frequency of amplifications of not only *MDM2* and *CDK4* but also other genes. Potential driver mutations were found in only six (11%) samples; however, gene amplification events (other than *MDM2* and *CDK4* amplification) were identified in 30 (54%) samples. Receptor tyrosine kinase (RTK) genes in particular were amplified in 18 (32%) samples. In addition, growth of a WDLPS cell line with *IGF1R* amplification was suppressed by simultaneous inhibition of CDK4 and IGF1R, using palbociclib and NVP-AEW541, respectively. Combination therapy with CDK4 and RTK inhibitors may be an effective therapeutic option for WDLPS/DDLPS patients with RTK gene amplification.

## INTRODUCTION

Liposarcoma (LPS) is the most common sarcoma of adults, accounting for 15%–25% of all soft tissue sarcomas (STSs). According to its clinicopathological and molecular genetic characteristics, LPS can be subdivided into three categories: well-differentiated/dedifferentiated, myxoid/round cell, and pleomorphic. Of these categories well-differentiated/dedifferentiated LPS (WDLPS/DDLPS) occurs most frequently (48–58% of all LPS) [1–3]. Surgical excision remains the standard of care for localized WDLPS/DDLPS, as these tumors are largely resistant to conventional cytotoxic chemotherapy. WDLPS is a locally aggressive neoplasm, classified as an intermediate malignancy virtually incapable of systemic spread. While lesions located in surgically amenable soft tissue do not recur after complete excision with a clear margin, tumors occurring in deep anatomical sites, such as retroperitoneum, tend to cause death as a result of uncontrolled local recurrence or dedifferentiation and subsequent metastasis. Overall, mortality rates range from 0% for WDLPS of the extremities to > 80% for WDLPS of the retroperitoneum after long term follow-up [1, 2, 4]. DDLPS is traditionally defined as a high-grade non-lipogenic sarcoma with a juxtaposed WDLPS area, and most commonly occurs in the retroperitoneum. Local recurrence is observed in ≥ 40% of all cases and in almost 100% of cases with retroperitoneal location. Distant metastases are observed in 15%–20% of cases and they are associated with considerably worse prognosis, with an overall mortality rate of 28%–56% at 5 year follow-up [1, 2, 4].

WDLPS and DDLPS exhibit similar cytogenetic features, characterized by giant marker and ring chromosomes containing amplified sequences of the 12q13–15 region, in which the *MDM2* (12q15) and *CDK4* (12q13–14) genes are amplified in 95%–97% and 85%–92% of cases, respectively [5, 6]. Amplifications of *MDM2* and *CDK4* cause their overexpression. MDM2 protein binds to p53 protein and stimulates p53 degradation; hence *MDM2* overexpression decreases apoptosis. CDK4 phosphorylates RB1 and prevents its interaction with the E2F transcription factor; hence *CDK4* overexpression allows the cell cycle to escape the G1–S checkpoint. Generally, DDLPS displays more extensive chromosomal abnormalities than WDLPS. The 12q13–15 amplifications in DDLPS are more complex than those in WDLPS. In addition, amplifications of other loci, including 1q23, 12q24, and either 6q23 or 1p32, are observed in approximately two-thirds of DDLPS cases. The *MAP3K5* gene in the 6q23 amplified region inhibits lipogenic differentiation through the JUN or PPARG-dependent pathways [7, 8].

In 2012, pazopanib was approved as the first molecular target drug for advanced STS on the basis of the results of the PALETTE study [9]; however, it did not demonstrate sufficient benefit in patients with LPS [10, 11]. Recently, small-molecule inhibitors of MDM2 and CDK4 (for example, RG 7112, flavopiridol, and PD0332991) have been developed and have shown promising results for the treatment of WDLPS/DDLPS in small-scale phase I and II clinical studies [12–16]. However, these drugs do not appear to be sufficiently effective as single agents on unresectable WDLPS and DDLPS; therefore, novel therapeutic targets are urgently needed for WDLPS/DDLPS.

Next-generation sequencing (NGS)-based genomic profiling of tumor tissues has contributed widely to the discovery of new therapeutic targets in many types of cancers [17, 18]. In addition, NGS-based targeted sequencing with small cancer-related gene panels has been used as clinical genomic testing for the selection of suitable molecular targeted therapies [19, 20]. The targeted sequencing of cancer-related genes enables rapid, highly sensitive, and cost-effective detection of actionable genetic alterations present in each tumor, including copy-number alterations; hence it is also an effective method for discovery of new therapeutic targets.

There are few reports of large-scale genomic profiling of WDLPS and DDLPS [21, 22]. Here, we performed targeted sequencing analysis of a relatively large cohort of 19 WDLPS and 37 DDLPS cases, using a panel of 104 cancer-related genes (NCC oncopanel v3), which was developed for genomic testing to select suitable molecular targeted therapies (Supplementary Table 1). We found that receptor tyrosine kinase (RTK) genes were amplified in approximately one-third of WDLPS/DDLPS samples and obtained data suggesting that inhibition of specific RTKs may become an effective therapeutic option for patients with tumors in which their genes are amplified.

## RESULTS

### WDLPS/DDLPS is characterized by few gene mutations and highly frequent gene amplifications

**Figure 1:**
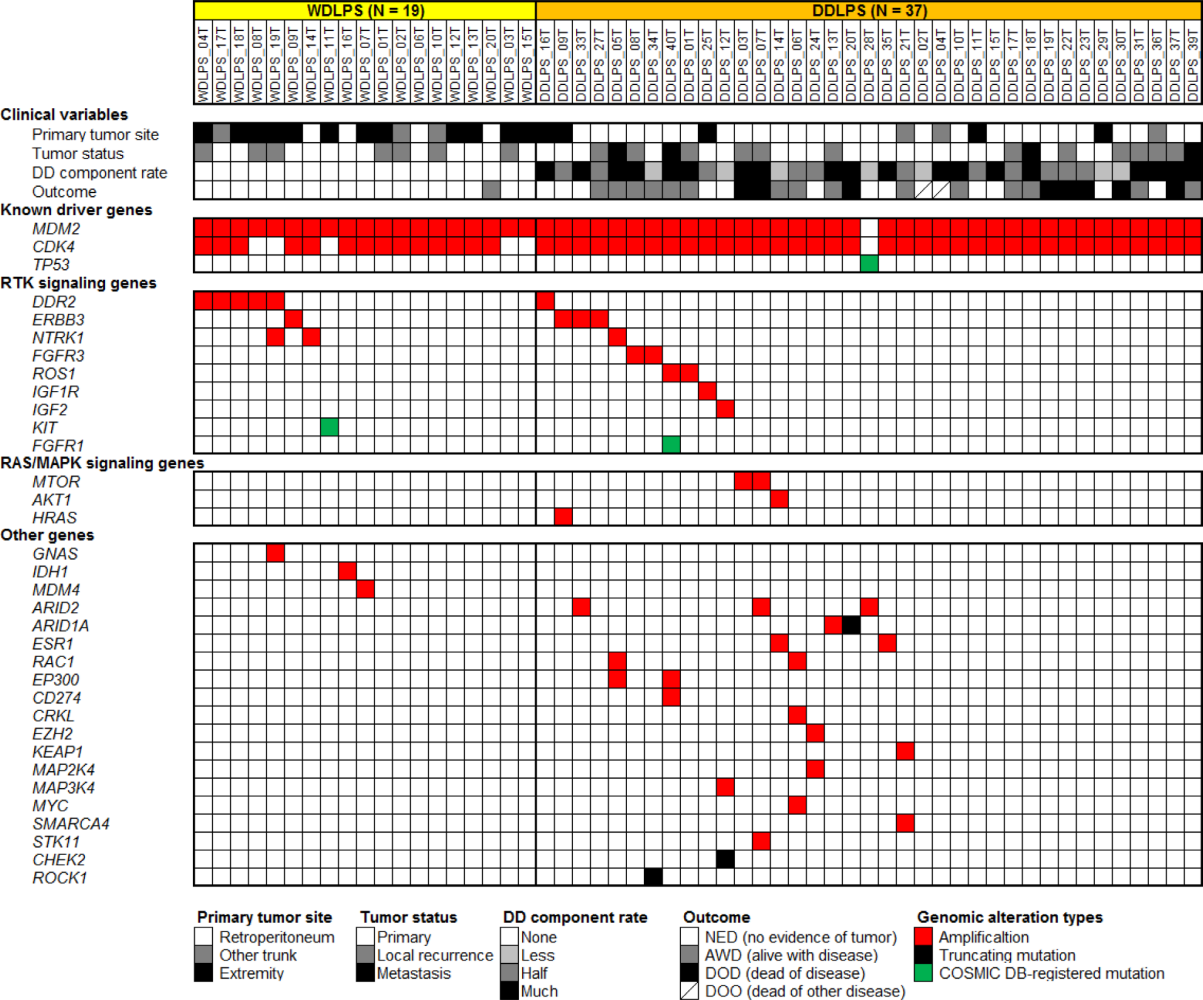
Summary of genetic alterations identified in 19 WDLPS and 37 DDLPS samples by targeted sequencing analysis using a panel of 104 cancer-related genes Each column represents a patient sample. The top section indicates clinical variables of each patient. The following three sections indicate genetic alterations found in each sample.

We analyzed 19 WDLPS and 37 DDLPS tumor tissue samples (Table 1) by targeted sequencing of 104 genes (Supplementary Table 1). All samples were histologically re-examined and their diagnoses were confirmed. Among them, one WDLPS (WDLPS_20T) and one DDLPS (DDLPS_6T) sample were derived from the primary and recurrent tumors, respectively, of the same patient. In this analysis, we did not examine paired normal control samples from the same patients. Instead, we obtained probable somatic mutations, by removing common single nucleotide polymorphisms (SNPs) registered in public and originally developed genomic sequence databases (see Materials and Methods). From this analysis, we identified a mean of 1.12 (1.05 in WDLPS and 1.16 in DDLPS) potential mutations (single nucleotide variations and short insertions and deletions) per patient after SNP elimination (Supplementary Table 2). When COSMIC database [23] registered mutations and truncating mutations were selected as those likely to be functionally important (potential driver mutations), the mean number of mutations decreased to 0.11 (0.05 in WDLPS and 0.14 in DDLPS) per patient (Figure 1 and Supplementary Table 2). These mutations occurred in the *TP53, KIT, FGFR1, ARID1A, CHEK2*, and *ROCK1* genes; no recurrently mutated genes were identified. By contrast, gene amplifications were frequently observed (Figure 1 and Supplementary Table 3). As expected, *MDM2* and *CDK4* were amplified in the majority of tumors; *MDM2* was amplified in 55 of 56 (98%) samples (19 of 19 WDLPSs and 36 of 37 DDLPSs), while *CDK4* was amplified in 50 (89%) samples (14 WDLPSs and 36 DDLPSs). Interestingly, the *MDM2* amplification-negative DDLPS sample had a TP53 missense mutation (Figure 1). In addition to the *MDM2* and *CDK4* genes, a mean of 0.79 genes (0.55 in WDLPS and 0.92 in DDLPS) were amplified per patient. The mean frequencies of potential driver mutations and amplifications were slightly higher in DDLPS (0.14 and 2.9 per patient, respectively) than in WDLPS (0.05 and 2.4 per patient, respectively).

**Table 1:** Clinical characteristics of WDLPS/DDLPS patients analyzed in this study

	Total (*N* = 56)	WDLPS (*N* = 19)	DDLPS (*N* = 37)
Age (years)			
Median (range)	60 (30–81)	60 (30–81)	60 (38–81)
Sex			
Male	42	12	30
Female	14	7	7
Tumor site			
Retroperitoneal	33	4	29
Other trunk	6	3	3
Extremity	17	12	5
Tumor size (cm)			
Median (range)	14.0 (3.0–38.0)	17.0 (6.5–38.0)	13.0 (3.0–31.0)
M0/M1			
M0	53	19	34
M1	3	0	3
TNM stage			
IA/IB	19	19	0
IIA/IIB	3	0	3
III	31	0	31
IV	3	0	3
Treatment			
Surgery only	45	19	26
Surgery + chemotherapy	3	0	3
Surgery + RT	3	0	3
PBT or CIRT only	2	0	2
Palliative therapy	3	0	3
Local recurrence			
No	30	18	12
Yes	26	1	25
Distant metastasis			
No	46	19	27
Yes	10	0	10
Recurrence			
No	29	18	11
Yes	27	1	26
Follow-up (months)			
Median (range)	42 (3–170)	29 (9–129)	62 (3–170)
Oncological outcome			
No evidence of disease	30	18	12
Alive with disease	16	1	15
Dead of disease	9	0	9
Dead of other cause	1	0	1

RT, radiation therapy; PBT, proton beam therapy; CIRT, carbon ion radiation therapy.

### Frequent amplification of RTK genes in WDLPS/DDLPS

Other than the *MDM2* and *CDK4* genes, the most remarkable category of amplified genes were those encoding RTKs, which were amplified in 18 of 56 (32%) samples (7 of 19 WDLPSs and 11 of 37 DDLPSs) (Figure 1). *DDR2* (1q23) was amplified in six (11%) samples (five WDLPSs and one DDLPS), *ERBB3* (12q13) in four (7%) samples (one WDLPS and three DDLPSs), *NTRK1* (1q23) in three (5%) samples (two WDLPSs and one DDLPS), *FGFR3* (4p16) and *ROS1* (6q22) in two (4%) samples (two DDLPSs), and *IGF1R* (15q26) in one DDLPS sample (2%). In addition to these RTK genes, *IGF2*, which encodes a ligand of the IGF1R and IGF2R receptors, was also amplified in one DDLPS sample (2%). Moreover, COSMIC database-registered mutations of *FGFR1* and *KIT* were also identified among our samples (Figure 1). These observations suggest that the activation of RTKs and their downstream signaling pathways plays an important role in WDLPS/DDLPS tumor development. Regarding other genetic aberrations, amplifications of *ARID2* (12p12), *MTOR* (1p36), *ESR1* (6q25), *RAC1* (7p22), and *EP300* (22q13) were recurrently observed (Figure 1).

**Figure 2:**
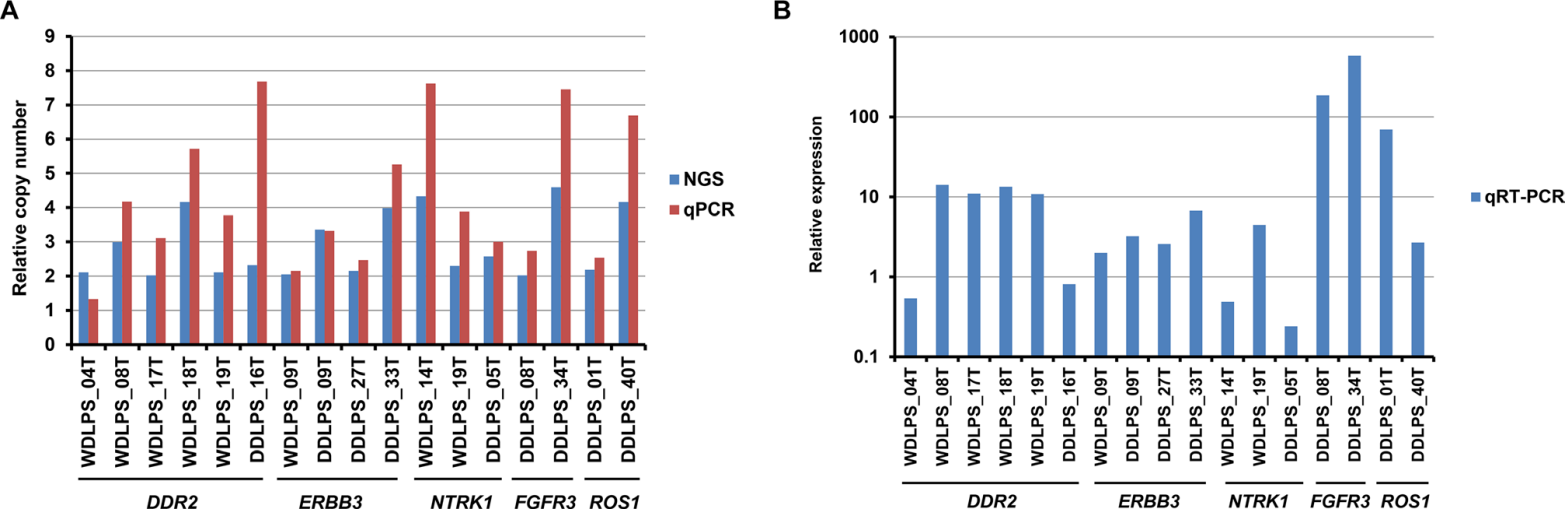
Quantitative PCR and RT-PCR analyses of recurrently amplified RTK genes (**A**) Comparison of NGS-estimated and quantitative PCR (qPCR)-estimated relative copy numbers of amplified RTK genes. (**B**) mRNA expression of amplified RTK genes estimated by quantitative RT-PCR (qRT-PCR), and normalized to GAPDH expression. Relative expression levels are expressed as ratios of the median expression in non-amplified samples.

Next, we performed quantitative PCR analysis to validate the amplification of RTK genes. We examined the five recurrently amplified RTK genes, *DDR2*, *ERBB3*, *NTRK1*, *FGFR3*, and *ROS1*, and all amplifications were confirmed (Figure 2A). While the quantitative PCR-estimated relative copy numbers were slightly lower than those estimated by NGS for two samples (WDLPS_04T and DDLS_09T), for the majority of samples they were higher (Figure 2A). The underestimation observed using NGS analysis was probably attributable to the relatively inefficient capture of highly amplified sequences with the limited amount of bait oligonucleotides used for the target enrichment process.

We also examined the mRNA expression of the recurrently amplified RTK genes. Quantitative RT-PCR analysis revealed that the amplified genes were overexpressed in 13 of 17 (76%) samples (Figure 2B), compared with the expression in non-amplified samples, and seven samples exhibited > 10-fold overexpression.

Then, we examined the clinical features of DDLPS patients with RTK gene amplification. Comparisons between the patients with RTK gene amplification (*N* = 11) and those without (*N* = 26) revealed no significant difference in any investigated clinical parameters, including patient age (*P* = 0.800), sex (*P* = 0.078), tumor status at presentation (*P* = 0.228), primary tumor site (*P* = 0.172), tumor size (*P* = 0.299), distant metastasis at presentation (M0/M1) (*P* = 0.240), TNM stage [24] (*P* = 0.220) (Supplementary Table 4), and disease-free survival (*P* = 0.402) (Supplementary Figure 1).

### Heterogeneity of RTK gene amplification

There are some differences in amplified loci between WDLPS and DDLPS. In our cohort, one DDLPS (DDLPS_06T) had developed as a local recurrent tumor from WDLPS (WDLPS_20T) 10 years after primary tumor resection. This case acquired additional amplification of the *RAC1*, *CRKL*, and *MYC* genes during the dedifferentiation process (Figure 1). In addition, we observed that gene amplifications were more extensive in DDLPS than in WDLPS samples (Figure 1).

**Figure 3:**
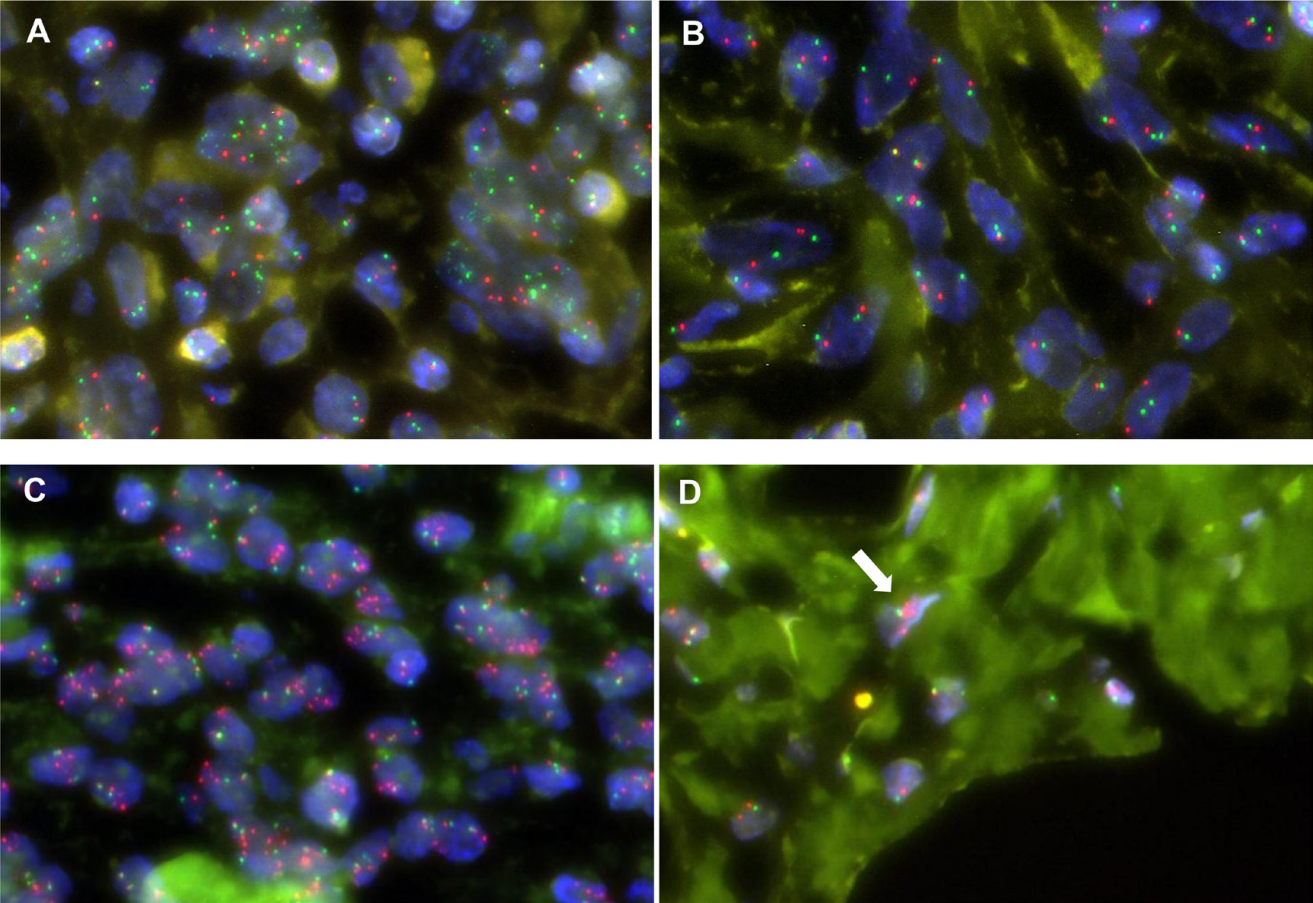
Intratumoral heterogeneity of RTK gene amplification in FISH Multiple FGFR3 signals (green) are observed in the majority of cells in a certain area (**A**) but not in any cells in another area (**B**) of the DD component of DDLPS_08T. Multiple ERBB3 signals (red) are observed in the majority of cells in the DD component (A) and only a few cells in the WD component (B) of DDLPS_27T.

DDLPS tumors exhibit histological heterogeneity, consisting of both well-differentiated (WD) and dedifferentiated (DD) components. To examine intratumoral spatial heterogeneity and further validate the RTK gene amplifications at the cell level, we performed fluorescence *in situ* hybridization (FISH) analysis of the *DDR2*, *ERBB3*, *NTRK1*, *FGFR3*, and *ROS1* genes in tumors in which they were amplified (seven WDLPSs and nine DDLPSs). The RTK gene amplifications in these 16 samples were repeatedly confirmed by FISH analysis (Table 2). The majority of cases had various degrees of intratumoral heterogeneity of RTK gene amplification (for example, see Figure 3A and 3B). In DDLPSs, RTK gene amplifications were generally enhanced in DD areas, compared with WD areas (Figure 3C and 3D and Table 2). These observations suggest that RTK gene amplifications are also involved in the progression from WD tumors to DD tumors.

**Table 2:** FISH analysis of recurrently amplified RTK genes

Gene	Sample	Amplification	
		DD area	WD area
*DDR2*	WDLPS_04T	NA	+
	WDLPS_08T	NA	++
	WDLPS_17T	NA	++
	WDLPS_18T	NA	++
	WDLPS_19T	NA	++
	DDLPS_16T	++	++
*ERBB3*	WDLPS_09T	NA	+
	DDLPS_09T	++	++
	DDLPS_27T	++	+
	DDLPS_33T	++	NS
*NTRK1*	WDLPS_14T	NA	++
	WDLPS_19T	NA	++
	DDLPS_05T	++	NS
*FGFR3*	DDLPS_08T	++	NS
	DDLPS_34T	++	NE
*ROS1*	DDLPS_01T	++	+
	DDLPS_40T	++	–

DD, dedifferentiated; WD, well differentiated; NA, not associated; NS, no sample, NE, not evaluable; “++”, positive in many tumor cells; “+”, positive in a small subset of tumor cells; “–”, negative.

To examine the heterogeneity of protein expression, we also performed immunohistochemistry analysis for the four proteins encoded by the RTK genes, *ERBB3*, *NTRK1*, *FGFR3*, and *ROS1*, in the 11 samples in which they were amplified. Unexpectedly, the majority of tumors produced negative staining results for these proteins, and only one DDLPS sample (DDLPS_08T) was very sparsely FGFR3-positive. In this sample, the cytoplasm of < 1% of tumor cells in the DD component was stained (Supplementary Figure 2). These results suggest that amplified RTK genes may only be expressed at the protein level in a limited population of tumor cells.

### Targetability of RTK gene amplification

**Figure 4:**
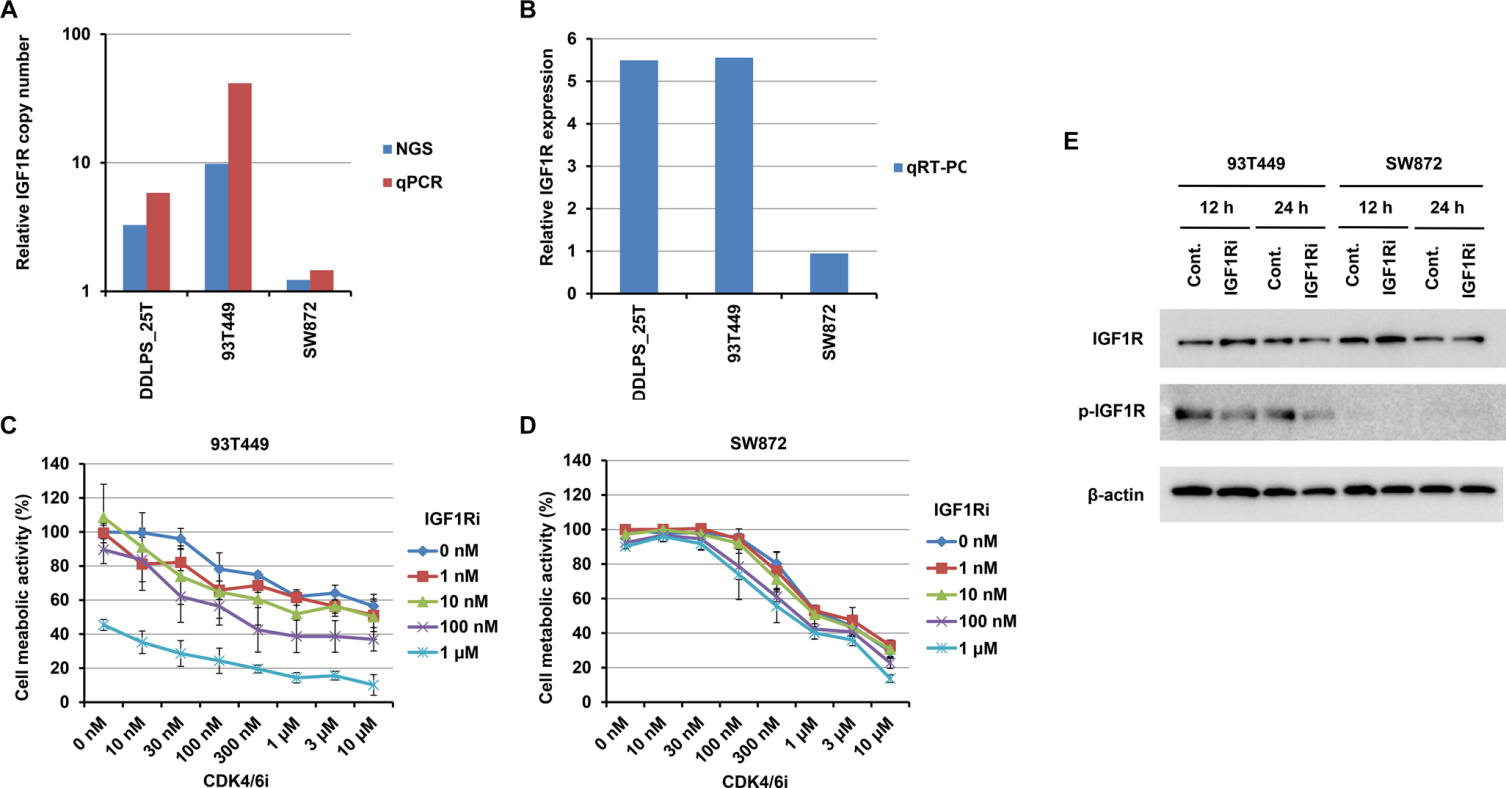
Effects on 93T449 and SW872 cells of combined treatment with CDK4 and IGF1R inhibitors (**A** and **B**) IGF1R amplification and expression in 93T449 and SW872 cells, as well as an IGF1R-amplified tumor (DDLPS_25T). Relative copy number was estimated by NGS and quantitative PCR (qPCR) (A). mRNA expression was estimated by quantitative RT-PCR (qRT-PCR) and normalized to GAPDH expression (B). Relative expression levels are expressed as ratios of the median expression in non-amplified tumor samples, as in Figure 2B. (**C** and **D**) Growth inhibitory effects of CDK4 and IGF1R inhibitors on 93T449 (C) and SW872 (D) cells. Palbociclib (CDK4 inhibitor) and NVP-AEW541 (IGF1R inhibitor) were added at various concentrations, and cell metabolic activities were assayed after 6 days of culture. In this assay, synergism of these inhibitors was evaluated using CompuSyn (http://www.combosyn.com) [41]. Their effects were synergistic in 93T449 cells (average combination index score = 0.42 ± 0.19), but not in SW872 cells (average combination index score = 6.28 ± 3.22). (**E**) Effect of IGF1R inhibitor on IGF1R phosphorylation in 93T449 and SW872 cells. Cells were treated with NVP-AEW541 (1 μM) for 12 or 24 h and harvested. Expression and Y1135 phosphorylation of IGF1R were evaluated by western blotting analysis.

Certain RTK gene alterations, such as *ERBB2* amplifications in breast cancer and *EGFR* mutations in lung cancer, are excellent therapeutic targets and used in clinical practice. To examine whether or not the RTK gene amplifications in WDLPS/DDLPS are therapeutically targetable alterations, we searched for WDLPS/DDLPS cell lines with RTK gene amplifications and found that a WDLPS cell line, 93T449, exhibited *IGF1R* amplification (Figure 4A) and overexpression (Figure 4B). Therefore, we performed growth inhibition assays using this cell line. This cell line was only slightly sensitive to the IGF1R inhibitor, NVP-AEW541, and the CDK4/6 inhibitor, Palbociclib (Supplementary Figure 3). Next, we tested a combination of these CDK4/6 and IGF1R inhibitors. Although single drug treatment with the CDK4/6 inhibitor (maximum concentration 10 μM) did not achieve 50% inhibition of 93T449 cell growth (Supplementary Figure 3), combined treatment synergistically reduced cell viability in a dose-dependent manner (Figure 4C). In IGF1R inhibitor-treated 93T449 cells, phosphorylation of IGF1R was decreased (Figure 4E). This synergistic effect using a combination of CDK4/6 and IGF1R inhibitors was not observed in the control unclassified liposarcoma cell line, SW872 (Figure 4D).

## DISCUSSION

In this study, we analyzed 19 WDLPS and 37 DDLPS tumor tissue samples by targeted genomic sequencing to identify targetable genetic alterations. Our results demonstrate that WDLPS/DDLPS harbored very few mutations and had a relatively high frequency of amplifications including of RTK genes. RTK gene amplifications were found in 18 of 56 (32%) samples, and *DDR2, ERBB3, NTRK1, FGFR3*, and *ROS1* were recurrently amplified.

Two large-scale genomic analyses of LPS tumors have previously been reported; however, they did not clearly describe the amplification of RTK genes in WDLPS/DDLPSs [21, 22]. Only one previous study has reported the identification of *DDR2* amplification as a novel therapeutic target by whole genome sequencing analysis of a single WDLPS tumor [25]. Therefore, to confirm our findings and estimate the prevalence of RTK gene amplification, we used cBioPortal for Cancer Genomics (http://cbioportal.org) [26, 27] to analyze data from two publically available sarcoma genome datasets: the dataset of the Memorial Sloan Kettering Cancer Center [21] and The Cancer Genome Atlas (http://cancergenome.nih.gov). Amplifications of the 20 RTK genes included in our gene panel were observed in 19 of 50 (38%) DDLPSs in the former dataset. Recurrently amplified RTK genes were *DDR2, NTRK1, ROS1, ERBB3, IGF1R, KIT*, and *PDGFRA* (Supplementary Figure 4A). In the latter dataset, the 20 RTK genes were amplified in 28 of 58 (48%) DDLPSs, with *ROS1, DDR2, ERBB3, FGFR4, IGF1R, NTRK1, FGFR1, FGFR3, PDGFRB*, and *RET* recurrently amplified (Supplementary Figure 4B). In addition, when 38 RTK genes not included in our panel were examined, we identified amplifications of *ROR1, INSR, AATK, INSRR, NTRK2*, and *FLT4* (Supplementary Figure 5). These similar observations in the three independent cohorts and the identification of additional RTK gene amplifications emphasize the functional importance of RTK gene amplification in WDLPS/DDLPS tumorigenesis.

Intratumoral heterogeneity can lead to underestimation of genomic alterations in the analysis of single tumor samples, and this can present major challenges to the development of personalized cancer medicine [28]. Therefore, we performed FISH analysis of the RTK gene amplifications identified by genome sequencing and found that their amplification exhibited varying degrees of intratumoral heterogeneity. In DDLPS tumors in particular, RTK gene amplification in DD areas was generally more extensive than that in WD areas. This result suggests that amplification of these RTK genes is also involved in tumor progression. If targeted therapy with inhibitors of these amplified RTK genes is effective, it is expected to be more beneficial in treating the more malignant DD areas than the WD areas.

Several agents targeting RTK genes have been investigated in preclinical or clinical situations for the treatment of WDLPS/DDLPS [29, 30]. In the preclinical studies, *EGFR, FGFR, MET, AXL, KIT*, and *IGF1R* were identified as overexpressed in WDLPS or DDLPS cells [31–33], and FGFR and MET inhibitors significantly inhibited the growth of DDLPS cell lines [31, 32]. By contrast, in clinical studies, drugs directed to RTKs, such as sorafenib (VEGFR and PDGFR inhibitor) and imatinib (ABL inhibitor), induced limited responses in phase II trials [34–36]. Pazopanib, an oral multi-targeted tyrosine kinase inhibitor with activity against VEGFR, PDGFR, and KIT, which has been approved as the first molecular targeting drug for advanced STS, also demonstrated an insufficient response in patients with LPS subtypes [10, 11]. However, in these studies, the genetic aberrations in each patient were not tested; therefore, the reported unsatisfactory results may be due to the use of drugs not matched to individual patients. Personalized therapy based on individual genomic alterations may increase response rates and lead to better clinical outcomes.

Our results, using a WDLPS cell line, 93T449, harboring co-amplification of *MDM2/CDK4* and *IGF1R*, indicate that matched RTK inhibitor treatment could be effective. Although this cell line was only slightly sensitive to a CDK4/6 inhibitor and an IGF1R inhibitor, combination treatment with both of these inhibitors dramatically improved efficacy (Figure 4C). The improvements observed with combination therapy are most likely because of cooperative inhibition of the multiple cellular signaling events typically altered in cancer [37, 38]. Interestingly, Miller *et al*. also reported CDK4 and IGF1R as synergistic drug targets in DDLPS cells (DDLS8817 and LPS141), using a drug synergy screen and network modeling approach [39]. As they already pointed out, CDK4 and IGF1R inhibitors probably function through the inhibition of different survival pathways (RB and AKT/mTOR, respectively).

In conclusion, we found that RTK gene amplifications are potentially targetable genetic alterations in WDLPS/DDLPS, present in between one-third and almost half of patients. For the WDLPS/DDLPS patients with RTK gene alterations, combination therapy with CDK4 and personalized RTK inhibitors could be effective. However, not all RTK gene amplifications led to overexpression of their mRNAs (Figure 2B), and expression at the protein level was also limited (Supplementary Figure 2B). To translate our findings to the clinic, it will be necessary to improve understanding of RTK gene amplification by more comprehensive genomic analyses, and to examine the significance of this phenomenon in additional cell lines and patient-derived xenograft models.

## MATERIALS AND METHODS

### Patient samples

WDLPS (*N* = 19) and DDLPS (*N* = 37) frozen tumor tissue samples from the National Cancer Center Biobank (Tokyo, Japan) were used. These tumor tissues were obtained from patients who underwent surgery at the National Cancer Center Hospital (Tokyo, Japan) between 1998 and 2013. Tumor samples were collected by pathologists from regions with high macroscopic tumor content immediately after surgical excision, and cryopreserved in liquid nitrogen until use. One patient with DDLPS (DDLPS_35) received neoadjuvant chemotherapy before surgery. All others did not receive chemotherapy or radiotherapy before surgery. The diagnosis of all tumors was confirmed by critical re-examination of the clinical and histopathological findings. Follow-up periods ranged from 3 months to 14 years, with a median of 3.5 years. Clinical characteristics of all patients are summarized in Table 1. This study was approved by the institutional review board at the National Cancer Center.

### Cell lines

Two human LPS cell lines, 93T449 (WDLPS) and SW872 (unclassified LPS), were used in this study. These cell lines were purchased from American Tissue Type Culture Collection (Rockville, MD, USA), and were maintained in RPMI-1640 medium supplemented with 10% fetal bovine serum (FBS) in a humidified incubator at 37°C with 5% CO_2_.

### Genomic DNA, RNA, and protein extraction

Fresh-frozen tissues were crushed to powder using a Multi-beads Shocker (Yasui Kikai, Osaka, Japan) under cooling with liquid nitrogen. Genomic DNA samples were extracted from frozen tumor tissue powder and cell lines using the standard phenol-chloroform extraction method. Total RNA was extracted from frozen tumor tissue powder using ISOGEN reagent (Nippon Gene, Tokyo, Japan) according to the manufacturer's protocol, and purified using an RNeasy MinElute Cleanup Kit (Qiagen, Hilden, Germany). The quality of total RNA was checked on a 2100 Bioanalyzer (Agilent Technologies, Santa Clara, CA, USA). Protein was extracted from cell lines using urea lysis buffer (6 M urea, 2 M thiourea, 3% CHAPS, and 1% Triton X-100). After centrifugation at 15,000 rpm for 30 min, the supernatant was used as the source of cellular proteins for western blotting analysis.

### Targeted sequencing analysis

For targeted sequencing analysis, an original gene panel, NCC oncopanel v3, developed specifically for genomic testing for the selection of suitable molecular therapy targets, was used. This panel was designed using SureDesign (Agilent Technologies, Santa Clara, CA, USA) to capture all coding exons of 104 genes and reported translocated introns of 16 genes (Supplementary Table 1). These genes were selected from the genes that were known to be somatically affected in solid tumors as of January 2014. Sequencing libraries were prepared using SureSelect XT reagent (Agilent Technologies). Paired-end sequencing (2 × 150 bp) was performed on MiSeq and HiSeq2500 sequencers (Illumina, San Diego, CA, USA).

To detect mutations (single nucleotide variations and short insertions and deletions), gene amplifications, and gene fusions from the sequencing read data, we used an in-house program cisCall (Kato M *et al*., manuscript in preparation). All detected alterations were checked by manual inspection. For SNP elimination, we used 1000 Genomes (http://www.1000genomes.org), ESP6500 (http://evs.gs.washington.edu/EVS/), Human Genetic Variation Database (http://www.hgvd.genome.med.kyoto-u.ac.jp/), and in-house Japanese germline SNP data. For annotation of identified mutations, we used ANNOVAR [40] and COSMIC [23] databases. COSMIC database-registered mutations in oncogenes, and COSMIC database-registered mutations and truncating mutations in tumor suppressor genes, were considered functionally important mutations (potential driver mutations). Increases in read depth > 2-fold were judged as gene amplification.

### Quantitative genomic PCR analysis

Quantitative PCR analysis was carried out using TaqMan Gene Expression Master Mix and TaqMan Copy Number Assays on a 7900HT Fast Real Time PCR System (Thermo Fisher Scientific, Waltham, MA, USA). TaqMan Copy Number Assays used for *DDR2, ERBB3, NTRK1, FGFR3, ROS1*, and *IGF1R* were Hs01066084_cn, Hs02182510_cn, Hs00946894_cn, Hs00136087_cn, Hs02890670_cn, and Hs02543373_cn (Thermo Fisher Scientific), respectively. The TaqMan Copy Number Reference Assay, human RNase P (Thermo Fisher Scientific), was used as a control. Genomic DNA (10 ng) was used as template for each PCR amplification.

### Quantitative RT-PCR analysis

Quantitative RT-PCR analysis was carried out using SYBR Premix Ex Taq II (Takara Bio, Kusatsu, Japan) on a 7900HT Fast Real Time PCR System. In addition to *DDR2, ERBB3, NTRK1, FGFR3, ROS1*, and *IGF1R, GAPDH* was evaluated as a control gene. PCR primers were designed using the Takara Perfect Real Time Support System (http://www.takara-bio.co.jp/prt/intro.htm) (Supplementary Table 5). cDNA was prepared from 100–500 ng of total RNA using Superscript III Reverse Transcriptase (Thermo Fisher Scientific), and a 1/100 amount of cDNA (corresponding to 1–5 ng of total RNA) was subjected to PCR amplification. The expression level of each gene was evaluated after normalization relative to *GAPDH* expression.

### Fluorescence *in situ* hybridization analysis

To validate amplification of the *DDR2, ERBB3, NTRK1, FGFR3*, and *ROS1* genes, fluorescence *in situ* hybridization (FISH) was performed on formalin-fixed, paraffin-embedded tumor samples. For DDLPS, samples containing WD and DD areas were selected. The probes used were *DDR2* (Texas Red)/CEN1p(FITC) FISH Probe (GSP Laboratory, Kobe, Japan) for *DDR2*, ZytoLight SPEC ERBB3/CEN12 Dual Color Probe (ZytoVision GmbH, Bremerhaven, Germany) for *ERBB3*, NTRK1(Texas Red)/CEN1p(FITC) FISH Probe (GSP Laboratory) for *NTRK1*, ZytoLight SPEC FGFR3/4p11 Dual Color Probe (ZytoVision) for *FGFR3*, and ZytoLight SPEC ROS1/CEN6 Dual Color Probe (ZytoVision) for *ROS1*.

### Immunohistochemistry analysis

Sections were exposed to 3% hydrogen peroxide for 15 min to block endogenous peroxidase activity and then washed with deionized water for 2−3 min. Heat-induced antigen retrieval was performed. The primary antibodies used were D22C5 (1:100, Cell Signaling Technology, Danvers, MA, USA) for ERBB3, EP1058Y (1:400, Abcam, Cambridge, UK) for NTRK1, B-9 (1:500, Santa Cruz Biotechnology, Santa Cruz, CA, USA) for FGFR3, and D4D6 (1:100, Cell Signaling Technology) for ROS1. Slides were incubated for 1 h at room temperature with the primary antibody and subsequently labeled using the EnVision system (Dako, Glostrup, Denmark). Diaminobenzidine was used as the chromogen, and hematoxylin as the counterstain.

### Western blotting analysis

Aliquots of protein samples (10 μg) were separated by SDS-PAGE. The separated proteins were subsequently blotted on to nitrocellulose membrane and incubated with primary antibodies against IGF1R (1:200, 7G11, Santa Cruz Biotechnology), Phospho-IGF-I Receptor β (Tyr1135) (1:1000, DA7A8, Cell Signaling Technology), and β-actin (1:5000, AC-15, Abcam, Cambridge, UK). The membrane was then treated with horseradish peroxidase-conjugated secondary anti-mouse antibody or anti-rabbit antibody (1:5000, Jackson ImmunoResearch Laboratories, Baltimore, PA, USA), processed using enhanced chemiluminescence reagents (Plus-ECL, PerkinElmer, Waltham, MA, USA), and scanned with a LAS-3000 laser scanner (FujiFilm, Tokyo, Japan).

### Cell viability assay

Next, the effects of the CDK4/6 inhibitor, Palbociclib (Selleckchem, Houston, TX, USA), and the IGF1R inhibitor, NVP-AEW541 (Selleckchem), on LPS cells were examined. The CCK-8 assay, which is widely used as a measure of cell metabolic activity, was performed according to the manufacturer's protocol (Dojindo Molecular Technologies, Kumamoto, Japan), after treatment with inhibitors. 93T449 and SW872 cells (1 × 10^4^) were plated in 100 μl of medium on 96-well plates and grown for 24 h. Cells were then treated with several different concentrations of single and combined drugs in triplicate by adding 100 μl of drug solution in medium. After 6 days of drug treatment, 10 μl of CCK-8 solution was added to each well and further incubated at 37°C for 3 h. Cell viability was determined by measuring the absorbance at 450 nm using a microplate reader (Tecan, Maännedorf, Switzerland).

### Statistical analyses

Statistical analyses were performed using the PASW Statistics 18 package (SPSS, Chicago, IL, USA). The significances of differences between two groups were evaluated with Student t and *χ*^2^ tests. Disease-free survival curves were plotted according to the Kaplan–Meier method, with the log-rank test applied for comparison. All differences at the level of *P* < 0.05 were considered statistically significant.

## SUPPLEMENTARY MATERIALS FIGURES AND TABLES








