# Supplementary material for: Frequent amplification of receptor tyrosine kinase genes in well-differentiated/ dedifferentiated liposarcoma
Source: Oncotarget. 2017 Jan 14;8(8):12941–52. doi: 10.18632/oncotarget.14652 (PMC5355068; doi:10.18632/oncotarget.14652)
Supplement: Supplementary file 1 [file oncotarget-08-12941-s001.pdf]

Frequent amplification of receptor tyrosine kinase genes in well-differentiated/dedifferentiated liposarcoma

Supplementary Materials

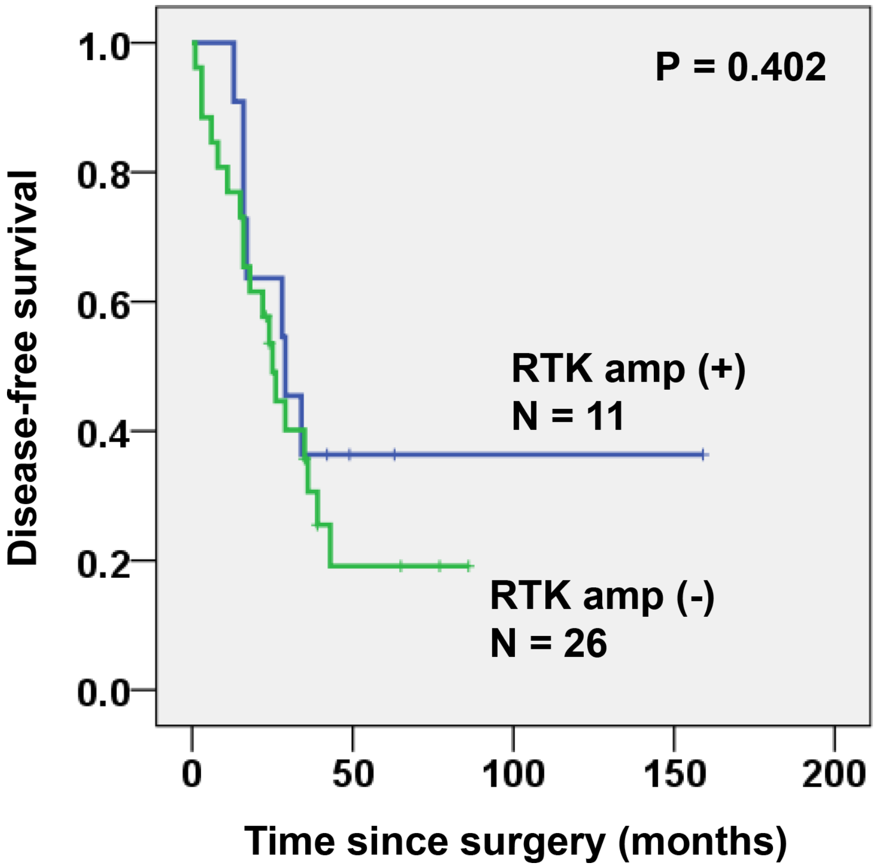

Supplementary Figure 1: Disease-free survival of DDLPS patients with and without RTK gene amplification. Disease-free survival of DDLPS patients was not significantly associated with RTK gene amplification (P = 0.402).

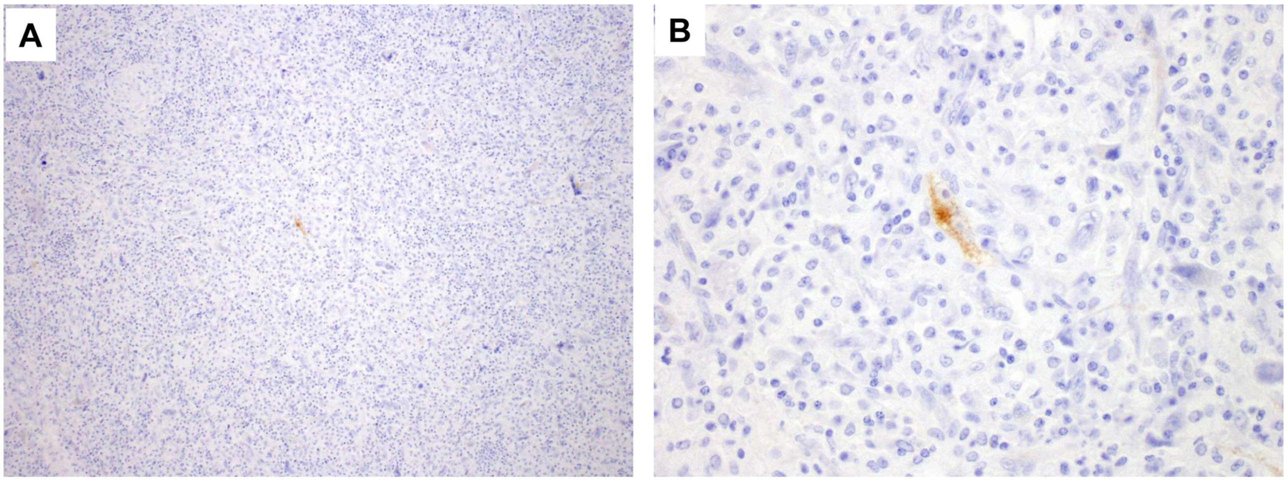

Supplementary Figure 2: Immunohistochemistry analysis of FGFR3 expression in DDLPS_08T. The cytoplasm of < 1% of tumor cells in the DD component was weakly stained under low-power (× 100) (**A**) and high-power (× 400) (**B**) magnification.

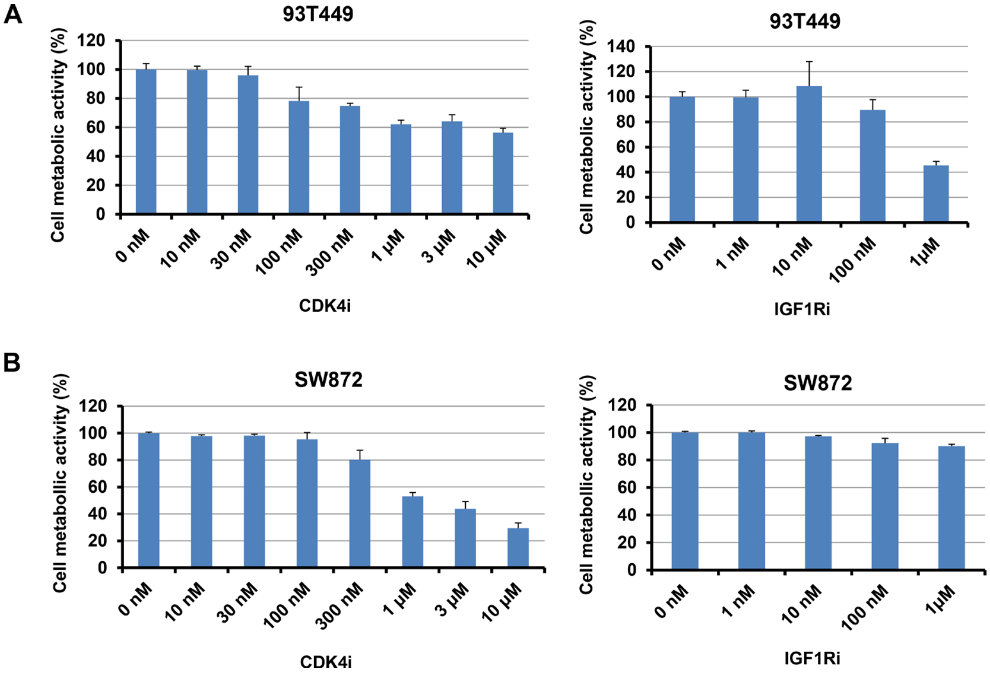

Supplementary Figure 3: Growth inhibitory effects of CDK4 and IGF1R inhibitors on 93T449 (**A**) and SW872 (**B**) cells Palbociclib (CDK4 inhibitor) and NVP-AEW541 (IGF1R inhibitor) were added at various concentrations, and cell metabolic activity was assayed after 6 days of culture.

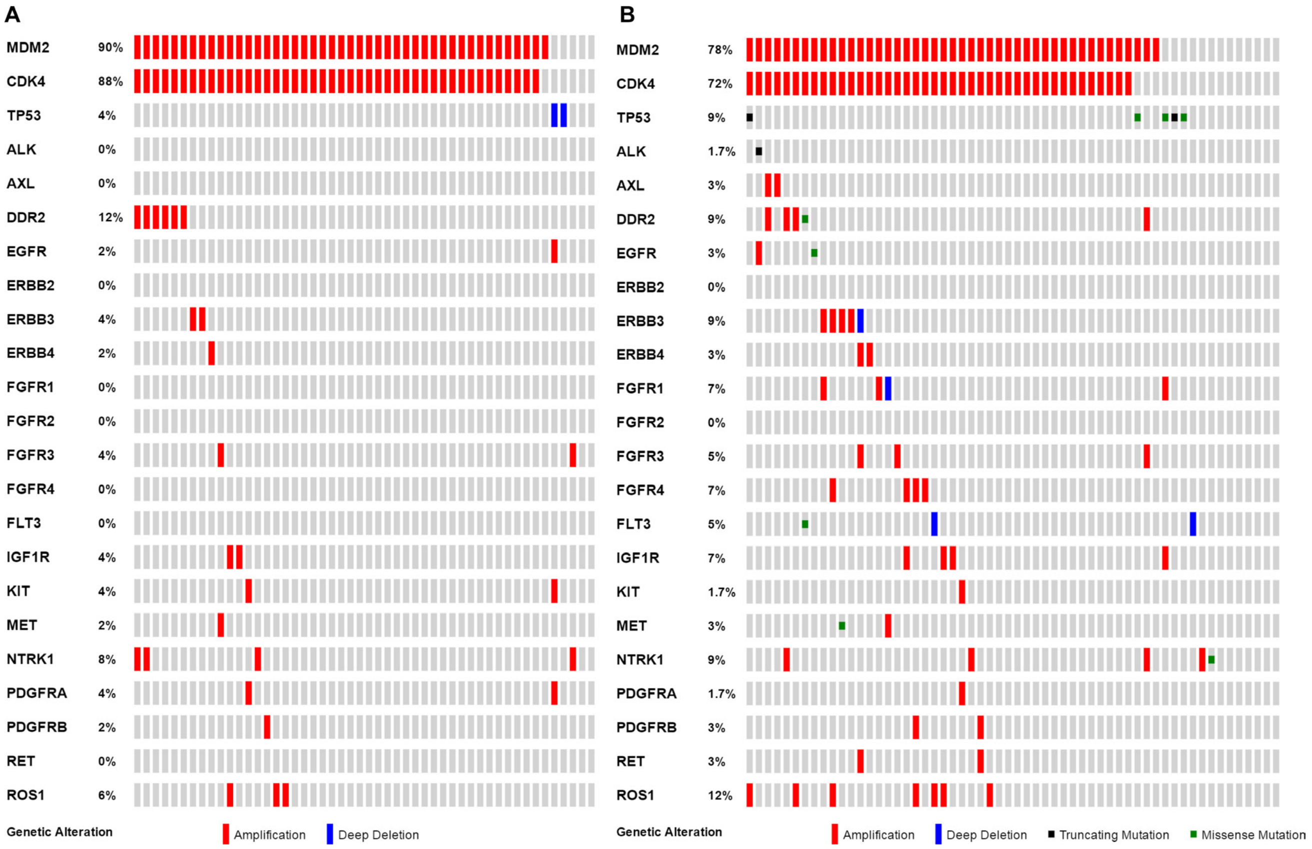

Supplementary Figure 4: Genetic alteration profiles of 20 RTK genes included in the gene panel in independent DDLPS cohorts. Genetic alterations of 50 DDLPS samples from the Memorial Sloan Kettering Cancer Center dataset (**A**) and those of 58 DDLPS samples from The Cancer Genome Atlas dataset (**B**) are summarized using cBioPortal for Cancer Genomics.

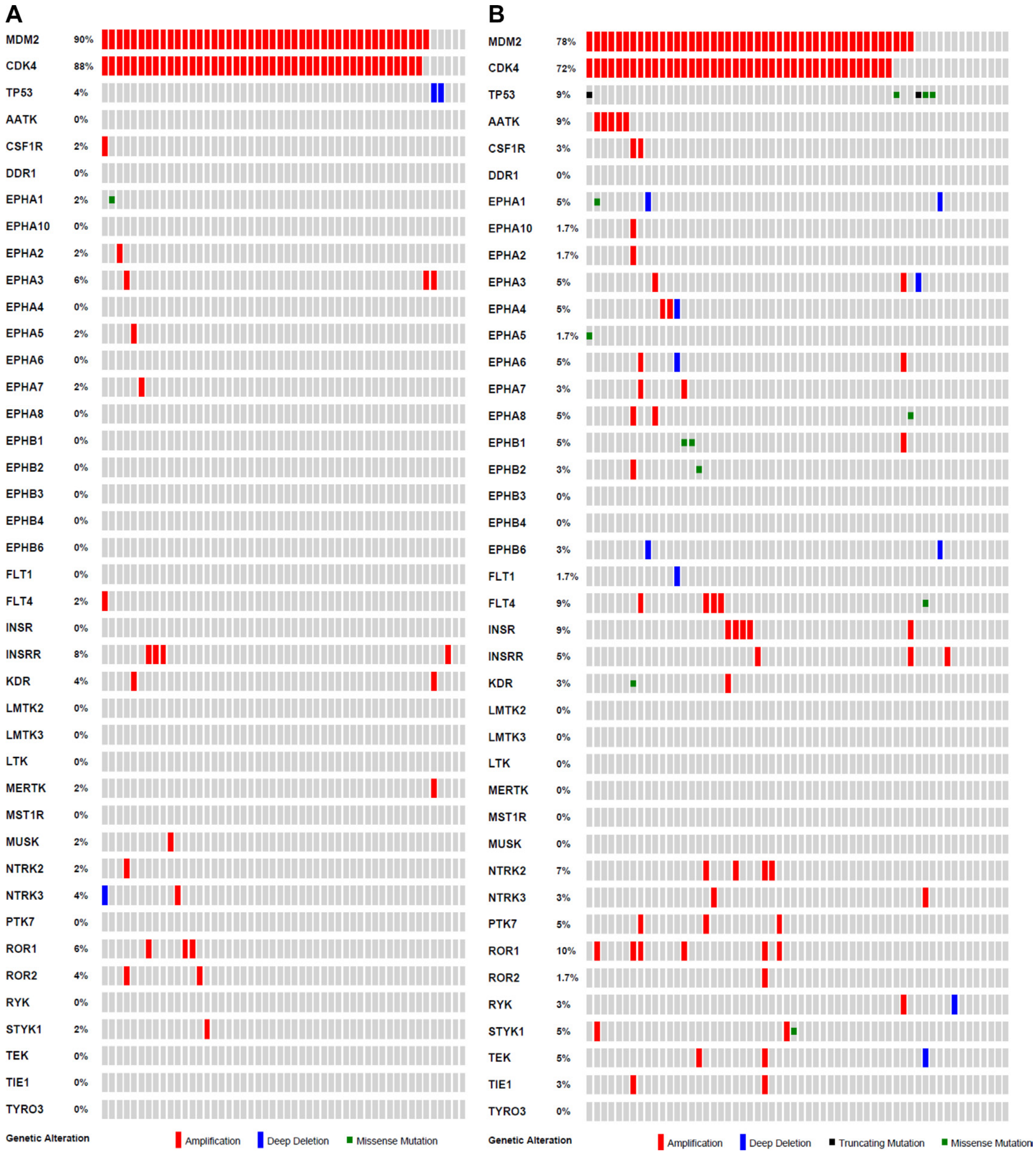

Supplementary Figure 5: Genetic alteration profiles of 38 RTK genes not included in the gene panel in independent DDLPS cohorts. Genetic alterations of 50 DDLPS samples from the Memorial Sloan Kettering Cancer Center dataset (**A**) and those of 58 DDLPS samples from The Cancer Genome Atlas dataset (**B**) are summarized using cBioPortal for Cancer Genomics.

Supplementary Table 1: NCC oncopanel v3 gene list. See Supplementary_Table_1

Supplementary Table 2: Probable somatic mutations identified in 19 WDLPS and 37 DDLPS samples. See Supplementary_Table_2

Supplementary Table 3: Gene amplifications identified in 19 WDLPS and 37 DDLPS samples. See Supplementary_Table_3

Supplementary Table 4: Clinical characteristics of DDLPS patients with and without RTK gene amplifications

	**Number of patients**			***P* value**
	**Total**	**Amplification (+)**	**Amplification (–)**	
Age				
< 60 years	19	6	13	0.800
≥ 60 years	18	5	13	
Sex				
Male	30	7	23	0.078
Female	7	4	3	
Tumor status				
Primary	25	9	16	0.228
Recurrent	12	2	10	
Tumor site				
Retroperitoneal	29	8	21	0.172
Other trunk	3	0	3	
Extremity	5	3	2	
Tumor size				
< 5 cm	3	0	3	0.299
≥ 5 cm/< 10 cm	10	2	8	
≥ 10 cm	24	9	15	
M0/M1				
M0	34	11	23	0.240
M1	3	0	3	
TNM Stage				
II	3	0	3	0.220
III	31	11	20	
IV	3	0	3	

Supplementary Table 5: PCR primers used in this study

Gene	Forward primer	Reverse primer
DDR2	CCCAGCTGTCAGATGAACAGGTTA	TCAGGACAAATGGCTGGTTGAG
ERBB3	TGCTGAGAACCAATACCAGACAC	AGCCTGTCACTTCTCGAATCC
FGFR3	TTTGGACTTCAAAGCAAGCTGGTA	TCTAATAACATCGGAACCTGCACAC
NTRK1	GACCCGGTGGAGAAGAAGGA	CATCCTCTGGAGCCAGCACA
ROS1	GGAAGTGGAGCCTTTGGAGA	AAACTCCAAGCTGCTTCAGAATG
IGF1R	GGTCTCTGAGGCCAGAAATGGA	TGGACGAACTTATTGGCGTTGA
GAPDH	GCACCGTCAAGGCTGAGAAC	TGGTGAAGACGCCAGTGGA
